# A High Grade Astrocytoma with Pilocytic Morphology in a 5-Month-Old American Bulldog

**DOI:** 10.3390/vetsci9100580

**Published:** 2022-10-20

**Authors:** Kelly Muller, Eunbee Kim, Abbie Lebowitz, Heather Daverio

**Affiliations:** Schwarzman Animal Medical Center, New York, NY 10065, USA

**Keywords:** MRI, dog, glioma, tumor, subdural hematoma, pilocytic, astrocytoma

## Abstract

**Simple Summary:**

Dogs are valuable models for spontaneous intracranial neoplasia in humans. The most common intracranial neoplasm in dogs is meningioma, followed by glioma and choroid plexus tumors. Pilocytic astrocytoma (PA) is a form of glioma and is a common childhood and adolescent tumor in humans. There have been two previous reports of PA in dogs. These cases were both adult dogs and the clinical and imaging features were not described. This report describes a 23-week-old female intact American Bulldog with a 2-week progressive history of neurologic dysfunction. Magnetic resonance imaging (MRI) revealed a large hemorrhagic mass that was confirmed on post-mortem examination to be a high-grade astrocytoma with pilocytic morphology. This case is the first to report a high-grade astrocytoma with pilocytic morphology in a juvenile dog.

**Abstract:**

A 23-week-old female intact American Bulldog was presented for a two-week history of progressive circling to the right, twitching, and altered mentation. Magnetic resonance imaging (MRI) revealed a non-contrast enhancing hemorrhagic mass centered in the right thalamus with concurrent subdural and intraventricular hemorrhage. Post-mortem histologic examination of the brain confirmed a mass centered on the thalamus with histomorphologic features consistent with a high-grade astrocytoma with pilocytic morphology. To the authors’ knowledge, the present case is the first to report clinical and imaging characteristics of a high-grade astrocytoma with pilocytic morphology in a young dog.

## 1. Introduction

The incidence of intracranial neoplasia in dogs is reported to be 14.5 in 100,000 [1,2]. Gliomas represent approximately 35 to 40% of all canine primary brain tumors [3,4]. Astrocytic tumors are the most common type of glioma and have been estimated to represent 17% of all primary central nervous system tumors [3]. Depending on the breed, brachycephalic dogs of similar lineage are 5 to 23 times more likely to develop a glioma compared to non-brachycephalic dogs [4,5]. A genetic component has been identified with a locus on canine chromosome 26 being strongly associated with glioma risk across multiple breeds [4,6].

Pilocytic Astrocytoma (PA) is a common pediatric tumor in humans, accounting for 15.4% of childhood and adolescent brain tumors [7,8]. In the revised 5th edition of the 2016 World Health Organization (WHO) Classification of Tumors of the Central Nervous System, PA is classified as WHO Grade I in the group “circumscribed astrocytic gliomas” [9]. This neoplasm is generally a well-circumscribed, slow growing tumor, with an overall 10-year survival rate of 90% [9,10]. Surgical excision is the treatment of choice, however, PA lesions that are unable to be fully resected may be treated with multi-modal therapy including radiation and chemotherapy [11,12]. A rare pilomyxoid variant has been reported that is thought to display more invasive growth and is classified as WHO grade II [9,13,14]. In veterinary medicine, a 2018 proposed revised classification scheme of canine glioma does not include PA [15,16]. Understanding of human tumor biology has been radically changed and improved by molecular and genetic testing; therefore, this new simplified classification scheme for canine gliomas hopes to serve as the basis for potential molecular and genetic characterization in the future [15,16]. Based on the revised scheme, the tumor in the reported patient represents a high-grade, infiltrative astrocytoma displaying pilocytic features. There is currently no reported treatment or prognostic information for PA in dogs, but a recent report showed longer median survival times with definitive (surgery, radiotherapy and/or chemotherapy) rather than palliative treatments in canine gliomas in general [16].

There are two previous reports of PA in dogs [17]. These two cases occurred in adult dogs and were confirmed on histopathology, but the reports did not include clinical or imaging findings. In this report, we describe the clinical and MRI characteristics of high-grade astrocytoma with pilocytic morphology in a young dog.

## 2. Case Description

A 23-week-old female intact American Bulldog was presented to the Emergency and Critical Care Department at the Schwarzman Animal Medical Center for evaluation of two weeks of progressive circling, twitching, hyporexia, hypodipsia, and diarrhea. Approximately four weeks prior to presentation, the patient had been relinquished to new owners. The progressive neurologic deficits were noted after the change in ownership. Prior medical history was unknown.

On presentation, the patient weighed 6.5 kg with a body condition score of 4 out of 9. Physical examination revealed hypothermia (temperature 95.5 °F), mild bradycardia (72 beats per minute), and moderate dehydration. The peripheral blood pressure was within normal limits. Comprehensive bloodwork was performed and revealed a severe hypernatremia (187 mmol/L; reference range 135–148 mmol/L) and severe hyperchloremia (value recorded as too high to read). Other blood work changes including the mild electrolyte, protein, and liver enzyme abnormalities were consistent with dehydration and the dog’s juvenile age. On neurologic examination, the dog was obtunded but responsive. She had a mild generalized ataxia and circled to the right. She displayed a right head turn, left head tilt, low head carriage, and fine head tremors. Cranial nerve examination identified a positional vertical to rotary nystagmus, absent menace in the left eye, and decreased nasal sensation on the left. Spinal reflexes and postural reactions were unremarkable. Neuroanatomic localization was multifocal with suspicion for right forebrain and left brainstem involvement, specifically the central vestibular system. Differentials for the patient’s neurologic signs included primary structural intracranial disease (congenital malformation, infection, neoplasia, neurodegeneration, immune mediated disease) or less likely metabolic/reactive etiologies. Due to the unknown duration and severity of the hypernatremia, it was elected to gradually correct the sodium elevation over three days prior to pursuing an MRI under general anesthesia.

The patient was hospitalized and started on a fluid treatment plan to correct the free water deficit over 72 h with 5% dextrose in water (D5W) (15 mL/hour) and Plasmalyte (maintenance rate of 60 mL/kg/day). The patient was also treated with Keppra (25 mg/kg IV every 8 h), Cerenia (1 mg/kg IV every 24 h), Metronidazole (15 mg/kg IV every 12 h), and Visbiome (1 capsule PO every 12 h). By the fourth day of hospitalization, the patient’s hypernatremia and hyperchloremia had significantly improved, but remained mildly elevated (154 mmol/L, 126 mmol/L respectively). Additionally, the patient was persistently hypothermic with only mild improvement in her diarrhea. Her neurologic examination remained unchanged despite supportive care and correction of her electrolyte abnormalities.

After three days of hospitalization, the patient was determined to be metabolically stable for anesthesia and an MRI was recommended. The dog was premedicated with IV administration of Butorphanol (0.2 mg/kg) and Maropitant (1 mg/kg). General anesthesia was induced with Midazolam (0.2 mg/kg IV) and Propofol (0.83 mg/kg IV) and maintained with the inhalant anesthetic isoflurane (0.75–1%). A constant rate infusion of dopamine (5–10 μg/kg/min) was utilized for blood pressure support due to persistent hypotension while under general anesthesia.

Magnetic resonance images of the brain were acquired using a 1.5 Tesla magnet and human knee coil (Achieva; Philips Medical Systems, Andover, MA, USA) with appropriate repetition time (TR) and echo time (TE) parameters. The study included T2-weighted images in sagittal and transverse (TR 4556, TE 85, 3.0 mm); pre- and post-contrast T1-weighted images in sagittal, transverse, and dorsal (TR 25, TE 6, 0.9 mm); a T2W-FLAIR sequence in transverse (TR 6600, TE 120, 3.3 mm); and a gradient echo (T2*) sequence in transverse (TR 1003, TE 9, 3.0 mm). The post contrast T1-weighted images were obtained after manual intravenous injection of the gadolinium-based contrast medium gadodiamide (Omniscan; GE Healthcare, Chicago, IL, USA).

**Figure 1:**
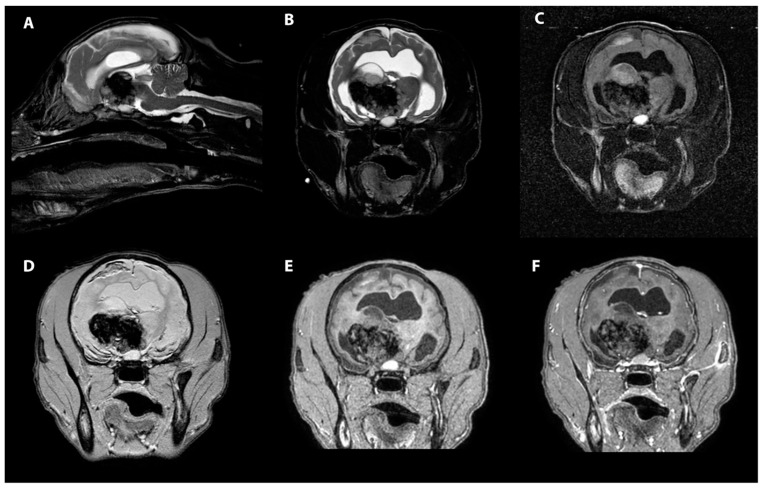
MRI images of the heterogenous intra-axial mass centered in the right thalamus in sagittal T2W (**A**), transverse T2W (**B**), transverse T2-FLAIR (**C**), transverse T2* (**D**), transverse T1W pre contrast (**E**) and transverse T1W post contrast images (**F**). The T1 and T2 hypointensity of the pituitary gland can be seen as well.

**Figure 2:**
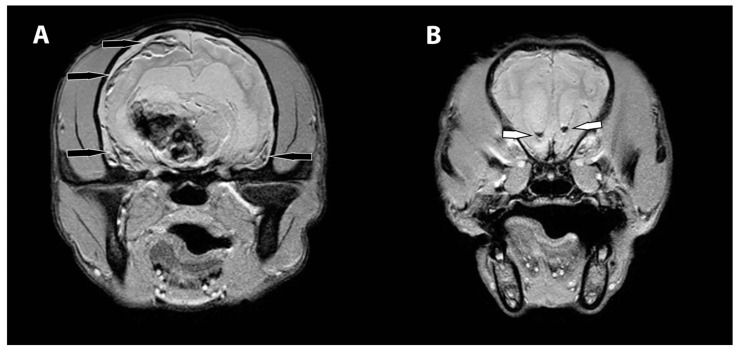
MRI images of the subdural and intraventricular hemorrhage. The transverse T2* weighted images (**A**) display heterogenous, mostly hypointense band of hemorrhage in the region of the subdural space, right worse than left (black arrows). More rostrally, the transverse T2* weighted images (**B**) show foci of signal void within the lateral ventricles, consistent with intraventricular hemorrhage (white arrows).

The MRI revealed an irregularly marginated, heterogenous, well circumscribed, non-contrast enhancing mass in the right thalamus and caudal right frontal lobe mostly displaying signal void on the T2 and T2* weighted images and T1W hypointensity (Figure 1). The dorsal aspect of the mass contained regions of T2- and T2*-weighted hyperintense and T1-weighted hypointense material. Secondary mass effect included leftward deviation and compression of the thalamus, mild compression of the right lateral ventricle, and mild caudal displacement of the midbrain with corresponding indentation of the rostral cerebellum. A mixed intensity band was noted between the dura mater and brain parenchyma along much of the dorsal and lateral cerebrum (most prominent ventrally), most consistent with extensive subdural hemorrhage. The meninges were diffusely contrast enhancing. The lateral and third ventricles were distended with a halo of FLAIR hyperintensity. Small foci of T2 and T2* signal voids were present in the rostral aspect of the lateral ventricles including the entire choroid plexuses, likely representing intraventricular hemorrhage (Figure 2). There was no evidence of peripheral trauma around the skull. The pituitary gland appeared rounded and strongly hyperintense on T1 and T2W images. Given the young age of the patient, consideration was given to a generalized coagulopathy or vasculitis associated with inflammatory or infectious etiologies. Trauma and neoplasia were considered less likely. The appearance of the pituitary gland was considered to be unrelated to the other findings and possibly incidental or benign. A coagulation panel, cerebrospinal fluid centesis, and infectious disease testing were offered for continued diagnostic work-up, but given the severity of the changes noted on the MRI and poor long-term prognosis, the owner declined further testing and elected humane euthanasia under general anesthesia.

**Figure 3:**
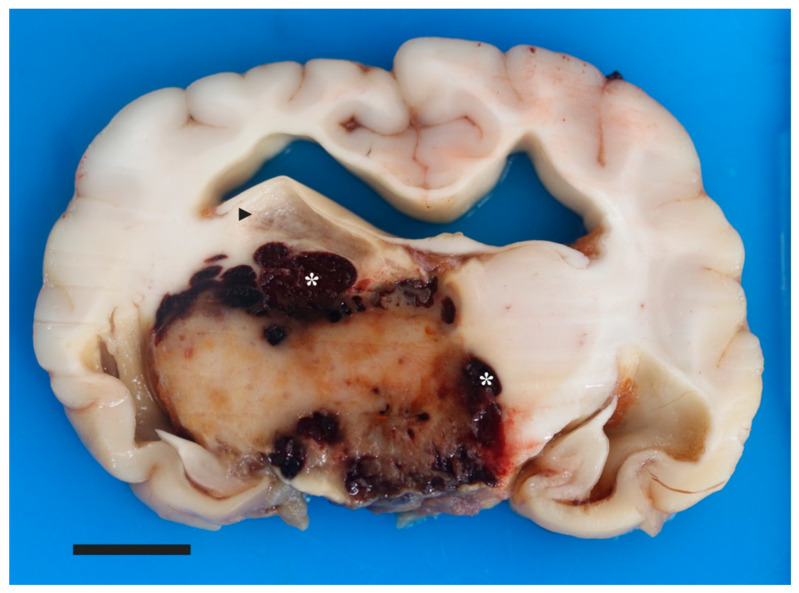
Pilocytic astrocytoma in a young dog’s brain. Macroscopic picture of cut surface of the brain at the level of the thalamus, depicting a well-circumscribed, 2.6 cm × 1.8 cm, expansile, tan/brown mass bordered by coalescing pockets of hemorrhage (asterisks). There is separation of the right fornix and stria habenularis from the dorsal aspect of the mass by a region of malacia (arrowhead). Moderate dilation of the lateral ventricles is depicted. Bar is 1 cm.

A postmortem examination was performed. Preliminary external brain evaluation on autopsy confirmed a large subdural hematoma. When viewed from the ventral surface, there was mild asymmetry of the diencephalon due to marked expansion of the right side and lesser expansion of the left side. Spanning from the region of the optic chiasm to the transverse fibers of the pons and bordered by the piriform lobes, the ventral aspect of the rostral perforated substance, optic chiasm, tuber cinereum, mamillary bodies, and crus cerebri contained dozens of coalescing areas of hemorrhage. The brain was fixed in 10% neutral buffered formalin for 7 days. Subsequently, the brain was serial sectioned after formalin fixation. Centered on the thalamus and spanning from the caudal frontal lobe to the level of the caudal piriform lobe was an approximately 2.6 cm × 1.8 cm (at its widest dimension), ovoid, firm, mottled tan, brown and red mass enveloped in hemorrhage (Figure 3). The surrounding neuroparenchyma was variably expanded and multifocally disrupted by gelatinous, semitranslucent, clear and light tan regions (malacia and edema). Additional findings included moderate hydrocephalus, mild to moderate intraventricular hemorrhage in the 3rd and 4th ventricles, and mild to moderate meningeal thickening suggestive of meningitis. Craniopharyngeal duct cysts were also identified in the pituitary gland, likely an unrelated incidental finding. Samples were routinely processed and 5 µm sections were stained with hematoxylin and eosin (H&E).

**Figure 4:**
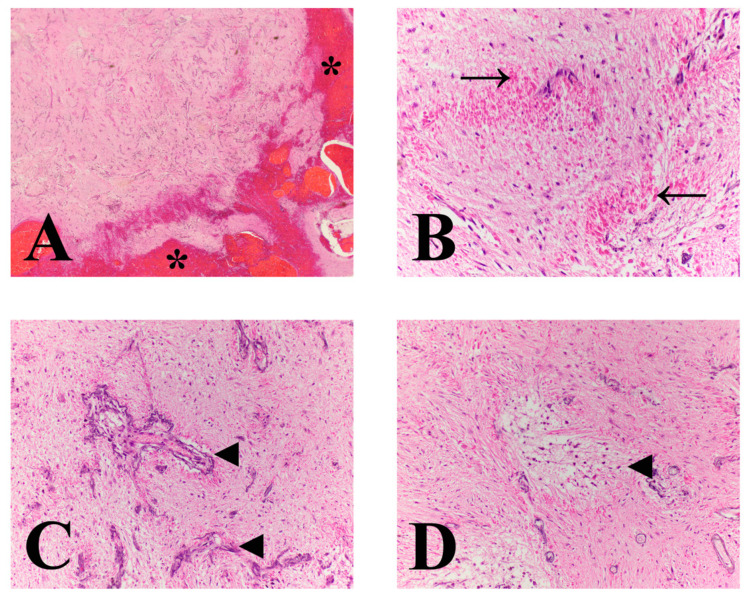
Microscopic features of a pilocytic astrocytoma in a dog. (**A**) Low power image at the mass interphase with mantling hemorrhage (asterisks). Mass is comprised of delicate, long fibrillated processes with numerous interspersed proliferating blood vessels. (**B**) Tumor displays long fibrillated appearance with numerous Rosenthal fibers (arrows). (**C**) Throughout the mass are numerous proliferating mineralized, tortuous and atypically branched blood vessels (arrowheads). (**D**) Neoplastic stroma is multifocally loose, separated, and vacuolated (arrowhead). The change is most prominent around blood. (**A**) magnification 20×; (**B**) and (**C**) magnification 400×; and (**D**) magnification 200×.

Histologically, the mass consisted of elongated bipolar cells with delicate streaming cytoplasmic processes on an eosinophilic fibrillary stroma with infrequent loosely arranged regions (Figure 4). Additional histomorphologic features of this mass included abundant Rosenthal fibers; numerous mineralized, tortuous and atypically branched blood vessels; tortuous blood vessels with piled and hypertrophied endothelial cells; perivascular hyalinized stroma; multifocal to coalescing areas of hemorrhage, edema, and individual cell necrosis; mononuclear inflammation with variable hemosiderin-laden macrophages and erythrophagia; and regions of parenchymal mineralization (Figure 4). No mitotic figures were identified in 10 [(40×) × 10×/FN22)] HPFs (2.37 mm^2^). The peritumoral neuroparenchyma was variably compressed and secondary tissue reactions included multifocal areas of parenchymal loss, hemorrhage, and edema; swollen axons (spheroids); Gitter cell infiltration; moderate gemistocytic astrocytes; moderate to severe neovascularization; and mononuclear perivascular cuffs. Further, histology confirmed subdural hematoma, intraventricular obstructive hydrocephalus, and histiocytic, lymphoplasmacytic, neutrophilic meningitis. Glial fibrillary acidic protein (GFAP) immunohistochemistry displayed diffuse and marked immunoreactivity within the mass. Based on the histomorphologic features, this tumor was classified as a high-grade astrocytoma with pilocytic morphology.

## 3. Discussion

Astrocytoma is the most commonly reported primary intra-axial tumor in dogs, and some brachycephalic breeds have a genetic predisposition [3,4,5]. Primary central nervous system tumors are most often diagnosed in middle-age to older dogs [3,4]. Though there have been previous reports of astrocytoma in young dogs, it is uncommon [18,19,20,21,22]. In humans, PA is most often a pediatric tumor, but has been reported in all ages [8,23]. In humans, PA is the most common primary brain tumor in pediatric patients (ages—0–19-years-old) and is associated with a single abnormality in the mitogen-activating protein kinase (MAPK) pathway [8,24]. DNA methylation patterns in pediatric and adult PAs in humans may suggest that infratentorial tumors are closely related, whereas those in the supratentorial brain may have age and location-specific differences [8]. Future investigation into the genetics of canine gliomas, including morphologic subtypes, may improve classification and prognostication. The two previously reported cases of PA in dogs were identified in adult dogs [17]. This case is the first report of a high-grade astrocytoma with pilocytic phenotype in a juvenile (23-week-old) dog.

The histopathology of the two previously reported cases of PA in dogs was very similar to the current case; the tumors contained fibrillated elongated cells; Rosenthal fibers; microcyst formation, and positive GFAP immunoreactivity [17]. These histopathologic features are also consistent with the typical features reported in cases of human PA [25].

The MRI findings in this case were unusual. Similar to the typical canine astrocytoma, the lesion was T1-weighted hypointense [26]. In contrast to a typical astrocytoma, this thalamic mass was mostly void of signal on T2 and T2*-weighted images with only small regions of T2-weighted hyperintensity, and was irregular in margination. A recent report found that poorly defined and irregular tumor margins had a higher death risk than smooth tumor margins in canine gliomas [16]. While lack of contrast enhancement of an astrocytoma, such as seen in this case, is less common, it has been reported. More strongly contrast enhancing astrocytomas usually represents higher grade tumors [26,27]. Additionally, there was a significant amount of intraventricular and subdural hemorrhage. While it is known that intratumoral hemorrhage can occur with canine gliomas especially those of higher grade, the extensive hemorrhage in the other regions of the brain was atypical [6,27]. Lastly, the generalized meningeal enhancement in this case was also an uncommon finding and likely represented secondary congestion or inflammation. Many of the features seen in this case were not expected for the typical canine high-grade astrocytoma. Further investigation into the imaging features of canine glioma subtypes is warranted.

In humans, the MRI features of PA have been well described. PA preferentially affects the cerebellum with other common locations including the optic pathway, hypothalamus, third ventricle, and brainstem. Less commonly, PA can be found in the cerebrum and spinal cord [10,28]. PA generally appears as a well demarcated, contrast-enhancing, T1-weighted hypo- or isointense, T2-weighted and FLAIR hyperintense lesion. A characteristic imaging feature of PA in humans is a cyst with an enhancing mural nodule [24,25,29]. Rarely, these tumors can resemble high-grade gliomas [30]. The MRI features of the PA in our case were vastly different from what has been described in the human literature.

The neuroanatomic localization in this case was multifocal, which is uncommon in cases with a solitary tumor, and was likely due to the hemorrhage, edema, and herniation secondary to the primary mass. The abnormal mentation, circling to the right, and right head turn were likely due to the right sided thalamic lesion. The hypothermia and hypodipsia were likely due to the secondary effects on the hypothalamus from hemorrhage and edema. The left head tilt, nystagmus, and ataxia were attributed to the increased intracranial pressure and secondary transtentorial herniation compressing the vestibular system. Tremors, which ultimately improved with correction of electrolyte derangements, were felt to be secondary to the hypernatremia, though a pain response cannot be entirely ruled out.

The severity of the hemorrhage in this case was striking and was likely due to the atypical tumor-associated blood vessels and encroachment on surrounding cerebral, meningeal, and potentially choroid plexus blood vessels. The intratumoral perivascular hemosiderin laden macrophages supported that the hemorrhage was ongoing within the tumor itself. There were additional regions of hemorrhage at the cranial-most aspect of the tumor (caudal frontal lobe) and the caudal-most aspect of the tumor (diencephalon/mesencephalon junction and region of the red nucleus). The hemorrhage in the 3rd ventricle with potential involvement of the choroid plexus may have led to more significant bleeding overall, however, invasion of the choroid plexus was not confirmed on histology. Additionally, there were regions where the tumor bordered or obscured choroidal and meningeal surfaces, suggesting potential encroachment of associated blood vessels, which may have led to the large subdural hematoma. The microvascular proliferation seen in this case is one criterion for increased grade. Other features of increased grade were not seen. Although rare, there have been cases reported in the human literature of PA lesions with significant internal hemorrhage [31].

## 4. Conclusions

Based on the clinical history, MRI findings, gross and histopathologic examination of the brain, this patient was diagnosed with a high-grade astrocytoma with pilocytic morphology. This is the first report of a pilocytic phenotype in a young dog, and the first report to describe the clinical and MRI characteristics. Neoplasia should be considered as a differential diagnosis of dogs with an intra-axial lesion of any age.

## Data Availability

Data are contained within the article.
